# Clinical recommendations for dry powder inhaler use in the management of COPD in primary care

**DOI:** 10.1038/s41533-022-00318-3

**Published:** 2022-12-27

**Authors:** Marika T. Leving, Sinthia Bosnic-Anticevich, Joyce van Cooten, Jaime Correia de Sousa, Biljana Cvetkovski, Richard Dekhuijzen, Lars Dijk, Marina Garcia Pardo, Asparuh Gardev, Radosław Gawlik, Iris van der Ham, Ymke Janse, Federico Lavorini, Tiago Maricoto, Jiska Meijer, Boyd Metz, David Price, Miguel Roman-Rodriguez, Kirsten Schuttel, Nilouq Stoker, Ioanna Tsiligianni, Omar Usmani, Rachel Emerson-Stadler, Janwillem W. H. Kocks

**Affiliations:** 1 General Practitioners Research Institute, Groningen, The Netherlands; 2 Woolcock Institute of Medical Research, University of Sydney, Sydney, Australia; 3 Sydney Local Health District, Sydney, Australia; 4 Life and Health Sciences Research Institute (ICVS), PT Government Associate Laboratory, School of Medicine, University of Minho, Braga, Portugal; 5 Radboud University Medical Center, Nijmegen, Netherlands; 6 Primary Care Respiratory Research Unit, Instituto De Investigación Sanitaria De Baleares (IdISBa), Palma de Mallorca, Spain; 7 Boehringer Ingelheim International GmbH, Ingelheim am Rhein, Germany; 8 Department of Internal Medicine, Allergology, Clinical Immunology, Medical University of Silesia, Katowice, Poland; 9 Department of Clinical and Experimental Medicine, Careggi University Hospital, Florence, Italy; 10 Faculty of Health Sciences, University of Beira Interior, Covilha, Portugal; 11 Observational and Pragmatic Research Institute, Singapore, Singapore; 12 Centre of Academic Primary Care, Division of Applied Health Sciences, University of Aberdeen, Aberdeen, United Kingdom; 13 Department of Social Medicine, Health Planning Unit, Faculty of Medicine, University of Crete, Rethymno, Greece; 14 Airway Disease, National Heart and Lung Institute (NHLI), Imperial College London and Royal Brompton Hospital, London, United Kingdom; 15 University of Groningen, University Medical Center Groningen, GRIAC Research Institute, Groningen, The Netherlands; 16 Dept. of Pulmonology, University of Groningen, University Medical Center Groningen, Groningen, The Netherlands

**Keywords:** Outcomes research, Chronic obstructive pulmonary disease

## Abstract

Over 1400 patients using dry powder inhalers (DPIs) to deliver COPD maintenance therapies were recruited across Europe and Australia. Their peak inspiratory flow (PIF) was measured, inhaler technique was observed, and adherence to treatment assessed. From relating the findings with patient health status, and thereby identifying critical errors, key clinical recommendations for primary care clinicians were determined, namely – measure PIF before prescribing a DPI to ensure inhalation manoeuvre ability is well-matched with the device. Some patients could benefit from inhalation training whereas others should have their DPI changed for one better suited to their inspiratory ability or alternatively be prescribed an active device (such as a soft mist inhaler or pressurized metered dose inhaler). Observing the inhalation technique was valuable however this misses suboptimal PIF (approaching one fourth of patients with a satisfactory observed manoeuvre had a suboptimal PIF for their DPI). Assess adherence as deliberate non-adherence can point to a mismatch between a patient and their inhaler (deliberate non-adherence was significantly associated with PIFs below the minimum for the DPI). In-person observation of inhalation technique was found to be inferior to video rating based on device-specific checklists. Where video assessments are not possible, observation training for healthcare professionals would therefore be valuable particularly to improve the ability to identify the critical errors associated with health status namely ‘teeth and lips sealed around mouthpiece’, ‘breathe in’ and ‘breathing out calmly after inhalation’. However, it is recommended that observation alone should not replace PIF measurement in the DPI selection process.

**Trial registration:** https://clinicaltrials.gov/ct2/show/NCT04532853.

## Introduction

The effectiveness of dry powder inhaler (DPI) maintenance therapy in chronic obstructive pulmonary disease (COPD) patients is associated with a complex constellation of factors, as assessed in the cross-sectional observational multinational PIFotal study^1,2^. This study found that suboptimal peak inspiratory flow (sPIF) and inhalation technique errors were associated with poor health status in COPD patients, whereas DPI adherence was not associated to poor health status^2^. According to the most recent International Primary Care Respiratory Group research prioritisation exercise^3^, significant evidence gaps remain within the study of respiratory diseases, coinciding with a lack of evidence-based guidelines, quality standards and training to support primary care for patients with respiratory diseases, such as COPD. Drawing on the views of primary healthcare professionals (HCPs) worldwide, there is a need for more effective clinical education in order to deliver best-practice primary care. With this in mind, we provide clinical recommendations from the PIFotal study – summarizing for primary care clinicians, a holistic approach to the clinical care of COPD patients using DPI maintenance therapies – taking into consideration PIF, inhalation technique and adherence to treatment.

The PIFotal study indicated that 29% of the COPD patients had insufficient inspiratory flow (a PIF lower than required for their DPI) during a typical day-to-day inhalation manoeuvre^2^. DPIs are breath-actuated devices and the amount of medication reaching the lungs depends upon the aerosol characteristics created by the patient’s inspiratory manoeuvre overcoming the internal resistance of the device and dispersing the dry powder medication, separating drug from carrier particles^4^. When the inspiratory effort is insufficient drug deposition in the lungs is reduced, compromising the effectiveness of the prescribed medication^4^. Therefore, for patients who exhibit insufficient inspiratory flow, there is a need to either deliver tailored instructions targeting at producing sufficient inspiratory flow^5^ or these patients should be switched to an alternative inhaler better suited to the patient’s inspiratory ability; also considering devices which do not depend upon a patient’s inspiratory ability, such as a soft mist inhaler (SMI), pressurized metered dose inhaler (pMDI), or a portable nebulizer. From a prescriber’s perspective, selecting a DPI fitting both patients’ needs and preferences is a complex decision, which should be reached together with the patient. However, among other factors such as inhalation technique, inhaler design and the environmental impact (including the plastic burden), PIF is one of the key factors to consider as sPIF can result in higher all-cause and COPD hospital readmissions^6^. It is known that inspiratory ability is compromised in COPD patients who experience exacerbations, and that exacerbations are associated with the inability to generate sufficient PIF for a DPI^7^. There is, however, little information about the best clinical practice to tailor individualised device selection vis-à-vis the necessity of taking PIF into consideration. Second, it is unclear whether it is important to measure PIF e.g., with portable assessment tools such as the In-Check DIAL G16 (Clement Clarke, UK) or whether observation of the inspiratory flow manoeuvre is sufficient to evaluate whether a DPI is well-matched to the patient.

Another important consideration in device selection is the patient’s ability, likelihood, desire to be adherent to their treatment regimen. A prior observational study found that deliberate non-adherence was linked to the inability to generate sufficient inspiratory flow for a DPI^8^. More importantly, patients with low PIF (<35 L/min) stopped using their inhaler more often than patients with sufficient PIF^8^. The association between PIF and adherence was therefore explored in PIFotal, where the medication adherence was poor in the overall study population^2^.

The PIFotal study revealed that inhalation technique errors were common and associated with worse health status^2^. In addition, a higher cumulative number of inhalation technique errors was associated with higher COPD-related healthcare costs^9^. These findings highlight the importance of correct DPI handling in managing COPD. Nevertheless, inhalation technique is often not assessed when selecting a DPI^10^, and there is currently no consensus about how such assessments should take place in daily clinical practice. Therefore, in this study we provide recommendations for clinical practice to assess inhalation technique in primary care.

## Methods

### Study design

The PIFotal study (clinicaltrials.gov identifier NCT04532853) was a cross-sectional observational real-world study in six countries (Australia, Greece, the Netherlands, Poland, Portugal, Spain)^1^. Patients were included in the study between October 2020 and May 2021. Local medical ethics committees reviewed and approved the study protocol, and all patients provided written informed consent. A description of the study procedures is available in Supplementary Fig. 1.

### Study population

A minimalist approach to the inclusion/exclusion criteria was used in order to ensure a real-world setting as much as possible. Patients were eligible for participation when clinically diagnosed with COPD, aged 40 years or older and treated with a DPI as maintenance therapy for their COPD in the previous 3 months or longer. Patients were excluded when they were unable to provide informed consent, were participating in other trials with COPD medication, experienced an exacerbation in the 6 weeks prior to participation, or had a life-threatening disease with a life expectancy <6 months.

### Peak inspiratory flow (PIF)

PIF (L/min) was objectively assessed with the In-Check DIAL G16 (Clement Clarke, UK), a multi-patient device set to resemble the internal resistance of the patient’s inhaler during an inhalation manoeuvre (with 6 settings of either a pMDI, low, medium low, medium, medium-high, or high resistance DPI device). If a patient used multiple DPIs for their maintenance therapy, a priority list based on the prevalence of DPIs in the participating countries (prioritising the more common inhalers to obtain the most representative and generalisable data) determined which DPI and corresponding internal resistance was used for the PIF assessment (Table S2)

PIF was assessed in three ways: 1) day-to-day typical PIF at the resistance of the patient’s DPI, 2) maximum PIF at the resistance of the patient’s DPI 3) maximum PIF at low internal resistance^2^. For the typical PIF measurement, participants were asked to inhale once with the In-Check DIAL G16 as they would normally do when using their DPI. For both maximum PIF measurements, participants were instructed to breathe out completely to empty the lungs, and then inhale as forcefully and fast as possible. Maximum PIF measurements were performed twice, and the highest PIF measurement was included in the data analysis. The following definitions of PIF were used in the analysis:

- ‘sPIF’: as typical PIF being lower than optimal for their device (for the cut-off values, Supplementary Table 1)
- ‘Low PIF’: as typical PIF being lower than minimally required for their device (for the cut-off values, Supplementary Table 1)

Furthermore, three clinically relevant groups were defined based on the PIF measurements:

- ‘Can and will do’: patients with a typical PIF ≥ than the optimal PIF for their device
- ‘Can, but will not do’: patients with a typical PIF below the optimal PIF for their device, but able to generate maximum PIF ≥ the optimal PIF
- ‘Cannot do’: patients with both their typical and maximum PIF < optimal PIF for their device

### Inhalation technique errors

Inhalation technique was observed and documented by video recording which was rated offline for errors by two independent observers. Checklists on inhaler-specific and inhaler-independent commonly made errors were used that were based on recommendations of the Netherlands Lung Alliance (www.inhalatorgebruik.nl) or, if unavailable for specific devices, the Aerosol Drug Management Improvement Team (www.inhalers4u.org). Differences between the two independent observers were resolved by discussion. In case non-consensus was reached, a third independent expert was consulted to reconcile the disagreement. The inhalation technique errors were dichotomous variables (‘yes’ / ‘no’ error observed). Inhalation steps marked as not applicable for the device were ‘no’ error. Inhalation technique was evaluated by grouping errors in steps together in 12 categories (Supplementary Table 2). For this study, we specifically focused on the errors ‘Breathe in incorrect’, ‘Teeth and lips sealed mouthpiece incorrect,’ and ‘Breathing out after inhalation incorrect’, as these errors were deemed ‘critical’ based on their frequency and significant association with worse health status in the PIFotal study^2^.

### Medication adherence

Adherence to the inhalers was assessed with the 12-item Test of Adherence to Inhalers^11^. Items 1 to 10 were answered by the patients and scored on a 5-point Likert scale (1–5). Item 11 was answered by the HCP performing the study visit. Due to precise offline assessments of inhalation technique, item TAI-12, concerning in-person clinician reported ‘critical’ inhalation technique errors, was replaced with the offline assessment of inhalation technique based on the video recordings. Of specific focus in this study were deliberate non-adherence, defined as TAI items 6 to 10 score <25, and item TAI-12 (i.e., the HCP assessed whether the patient revealed a critical inhalation technique error or if inhalation technique was correct during the visit).

### Health status

COPD-related health status was measured with the 10-item self-administered Clinical COPD Questionnaire (CCQ), consisting of three domains: symptoms, functional status, and mental health^12^. The CCQ score is the mean score of 10 item-scores, where each item is scored on a 7-point Likert scale (0–6), with higher scores indicating worse health status.

### Statistical analyses

Patient characteristics (including demographic variables, medication regimen, comorbidities) and PIF, adherence, inhalation technique error frequency and health status were described using descriptive statistics.

In order to define clinical practices aimed to optimise the use of DPIs, the following analyses were conducted, and recommendations proposed:

1. The proportion of patients within the three clinically relevant groups (can and will do, can but will not do, cannot do) was identified, and to simulate the impact of an exacerbation, the proportions were derived following a 20% reduction in a typical and maximal PIF^13^.
2. The association between the objectively measured sPIF with the In-Check DIAL G16 (Clement Clarke, UK), the observed error ‘Breathe in’ and health status (CCQ) was assessed with a linear regression model adjusted for potential confounders (Supplementary Table 3). Patients were categorized into four groups based on their PIF (optimal or suboptimal) and whether the error ‘Breathe in’ was observed (yes/no), which was regressed on the clinical outcome CCQ.
3. The association between deliberate non-adherence and low PIF (below the minimum flow for the device) was assessed with a logistic regression model adjusted for potential confounders (Supplementary Table 3). The odds-ratios (OR) and 95% confidence interval (95% CI) of having a low PIF was compared for deliberate non-adherent (TAI items 6 to 10) and adherent patients.
4. The agreement between clinician reported ‘critical’ inhalation technique (TAI item 12) and ‘critical’ error rating based on the video recordings using checklists was determined.

A sample size calculation was performed before study execution for the main study objectives^1,2^, and not specifically for these post-hoc analyses defined to deduce clinical recommendations from the PIFotal study. All statistical analyses were performed using Stata version 17.

The PIFotal COPD study protocol received approvals from the following institutional ethics committees/institutional review boards: Australia: Human Research Ethics Committee (HREC 3) University of Sydney; Greece: Research Ethics Committee University of Crete; Poland: Komisja Bioetyczna przy Beskidziej Izble Lekarskiej – Bielsko Biala; Komisji Bioetycznej przy Śląskiej Izbie Lekarskiej; Silesian Medical Society (Śląska Izba Lekarska); Bioethics Committee at Lower Silesian Medical Association; Bioethics Committee at the Medical University of Biaystok; Portugal: North Health Regional Administration (ARS Norte); Matosinhos Local Health Unit (ULS Matosinhos); Guimarães Hospital; Center Health Regional Administration (ARS Centro); Regional Health Administration of Lisbon and Tagus Valley (ARS LVT); Spain: Comité de Ética de la Investigación (CEI) Islas Baleares; CEI Hospital Universitario de Gran Canaria; The Netherlands: Medisch Ethische Toetsingscommissie (METC) Assen exempted this study.

### Reporting summary

Further information on research design is available in the Nature Research Reporting Summary linked to this article.

## Results

### Study population

A total of 1434 patients were included in the study and provided signed informed consent. An overview of the selection of the study population can be found in Supplementary Fig. 2.

Patient characteristics are shown in Table 1. Of these patients, 50.1% were female and the mean (SD) age was 69.2 (9.3) years.

**Table 1:** Overview of patient characteristics.

Variable		Total (n = 1434)
Age (years)	Mean (SD)	69.2 (9.3)
Sex	Male, *n* (%)	716 (49.9)
	Female, *n* (%)	718 (50.1)
Body Mass Index (kg/m^2^)	<18.5, *n* (%)	22 (1.5)
	18.5-<25, *n* (%)	432 (30.1)
	≥25-<30, *n* (%)	562 (39.2)
	≥30-<40, *n* (%)	382 (26.7)
	≥40, *n* (%)	35 (2.4)
Smoking status	Current, *n* (%)	436 (30.4)
	Former, *n* (%)	824 (57.5)
	Never, *n* (%)	174 (12.1)
Medication class in primary inhaler	LABA, *n* (%)	112 (7.8)
	LAMA, *n* (%)	385 (26.8)
	LABA/LAMA, *n* (%)	357 (24.9)
	LABA/LAMA/ICS, *n* (%)	63 (4.4)
	ICS, *n* (%)	9 (0.6)
	ICS/LABA, *n* (%)	506 (35.3)
	Short-acting, *n* (%)	2 (0.1)
GOLD stage^a^	*n* (% non-missing)	801 (55.9)
	I, *n* (%)	189 (23.6)
	II, *n* (%)	440 (54.9)
	III, *n* (%)	139 (17.4)
	IV, *n* (%)	33 (4.1)
Clinical COPD Questionnaire (CCQ)	Mean (SD)	1.7 (1.1)

^a^This variable missed data, *n* (%) of non-missings are reported.

#### The proportion of patients with suboptimal PIF by device resistance, and potential interventions based on PIF

**Fig. 1:**
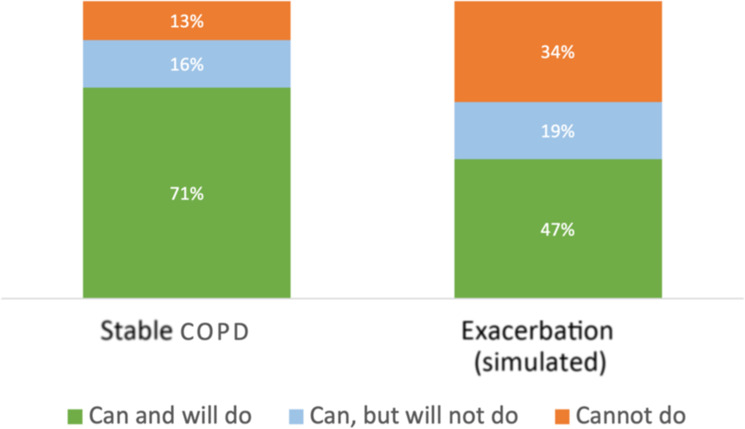
Distribution of clinical groups of inspiratory ability, based on measured PIF at stable COPD (left) and simulated 20% reduction in PIF during an exacerbation (right)^13^. ‘Can and will do’: patients with a typical PIF ≥ than the optimal PIF for their device; ‘Can, but will not do’: patients with a typical PIF below the optimal PIF for their device, but able to generate maximum PIF ≥ the optimal PIF; ‘Cannot do’: patients with both their typical and maximum PIF < optimal PIF for their device.

PIF measurements were available for 1389 patients. 71% (*n* = 987) of the patients were able to generate sufficient inspiratory effort for their device (‘can and will do’), whereas 16% (*n* = 219) were able to generate sufficient inspiratory effort but failed to do so (‘can, but will not do’), and 13% (*n* = 183) of the patients were revealed insufficient inspiratory effort (‘cannot do’) (Fig. 1, left).

In a scenario resembling the compromised PIF during an exacerbation, with a 20% reduction of the typical and the maximum PIF^13^, 24% of the total population who were initially in the ‘can and will do’ PIF category, would potentially not be able to perform sufficient inspiratory effort to perform a proper inhalation manoeuvre. Specifically, 62% of the patients in the ‘can, but will not do’ group would be categorized as ‘cannot do’ because of an exacerbation. Thus, 34% of all patients in this study would not be able to perform a proper inhalation with their current device during an exacerbation, compared to 13% of patients who were not able to do this under ‘usual’ conditions (Fig. 1), Supplementary Table 4).

Patients in the ‘can and will do’ PIF category generated sufficient inspiratory effort for their device, however, regular assessments of PIF are recommended to ensure consistent inspiratory effort over time. In addition, the impact of exacerbations (Fig. 1) should be considered for these patients (a quarter of patients could be compromised during an exacerbation).

Patients in the ‘can, but will not do’ PIF category, who were able to generate sufficient inspiratory effort for their device but failed to do so, could benefit from inhalation technique training to improve their device use. Switching to an alternative DPI (first checking if the patient can achieve the optimal PIF required) or an active device (SMI or pMDI) could be considered particularly if the patient has a higher exacerbation risk.

**Fig. 2:**
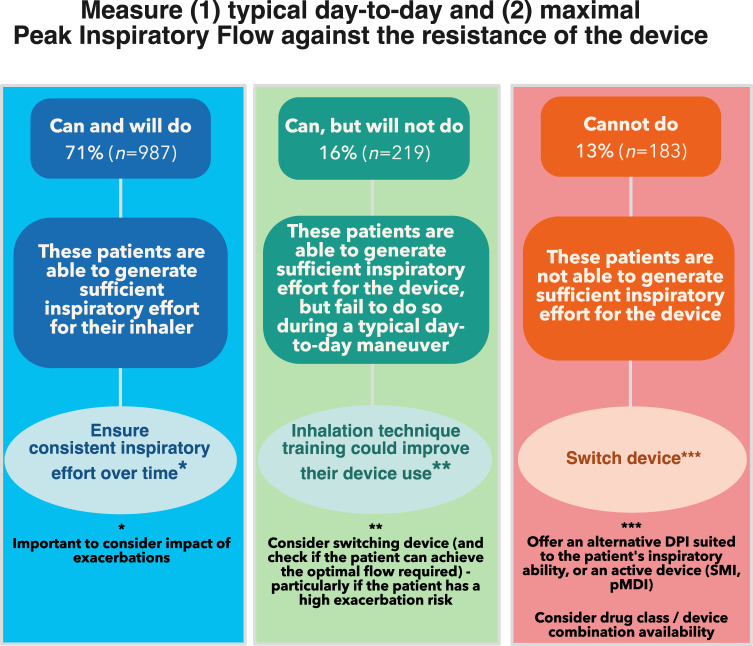
Decision tree for matching the DPI to the patient with COPD based on Peak Inspiratory Flow. ‘Can and will do’: patients with a typical PIF ≥ than the optimal PIF for their device; ‘Can, but will not do’: patients with a typical PIF below the optimal PIF for their device, but able to generate maximum PIF ≥ the optimal PIF; ‘Cannot do’: patients with both their typical and maximum PIF.

Patients in the ‘cannot do’ group could benefit from a different inhaler; either an alternative DPI better suited to the patient’s inspiratory ability, or an active device (SMI or pMDI) (Fig. 2).

#### Association between suboptimal PIF measured with an In-Check Dial G16 and the observed ‘Breathe in’ error with health status

**Fig. 3:**
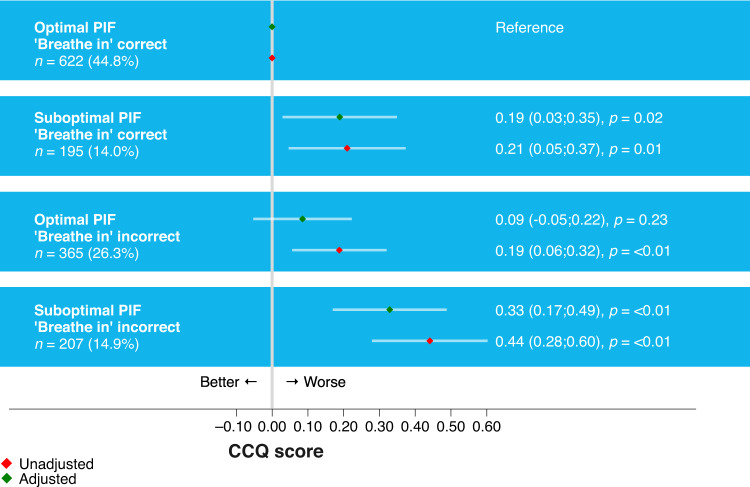
Association of PIF and ‘Breathe in’ error with health status (CCQ score). Regression model adjusted for the following confounders: Country of residence, Anxiety, Medication Regimen, Smoking status, Device resistance, BMI, Diabetes, Sex.

In 44.8% of the patients, both the measured PIF was optimal, and the observed inspiratory manoeuvre was correct. 14.9% of the patients exhibited both an sPIF and were observed to have an ‘Breathe in’ technique error (Fig. 3). In 40.3% of the patients, there was a discrepancy between the objective PIF assessment and the observed ‘breathe in’ error.

Even when PIF was observed to be sufficient, as assessed with video (*n* = 817), 24% of patients (*n* = 195) had sPIF, as objectively measured with In-Check G-16. Notably, patients with the ‘breathe in’ step scored as correct with video, but with sPIF objectively measured with an In-Check Dial G-16, had significantly worse health status compared to patients with optimal PIF and sufficient inspiratory effort based on the video (CCQ score 0.19; 95 % CI [0.03, 0.35]; *p* = 0.02) (Fig. 3).

As observation alone does not accurately identify patients with sPIF (a factor associated with significantly worse health status^2^). The recommendation therefore would be to measure PIF objectively (where possible) and follow the decision tree in Fig. 2.

#### Association between deliberate non-adherence and low PIF

Circa 8% of the patients had a low PIF (below the minimal PIF value required for their device, Table 2). Deliberately non-adherent patients were almost twice as likely to have a low PIF compared to those who were adherent (OR 1.94, 95% CI [1.26, 3.00] *p* = <0.01; Table 2).

**Table 2:** The association between deliberate non-adherence and low Peak Inspiratory Flow.

Resistance cluster	*N*	*N* (%) low PIF (i.e., typical PIF < minimal PIF for device)
Low	317	50 (15.8)
Medium low	371	19 (5.1)
Medium	359	25 (7.0)
Medium High	103	6 (5.8)
High	239	11 (4.6)
Total	1389	111 (8.0)
Predictor	Odds Ratio for low PIF (typical PIF < minimal PIF) Unadjusted	Adjusted^a^
Deliberate non-adherence (vs adherent)	1.76 [1.17, 2.64] *p* < 0.01	1.94 [1.26, 3.00] *p* < 0.01

^a^Confounders retained in the model: Device Resistance, Age, Inhalation technique error: ‘Hold breath’, ‘Anxiety’.

It is therefore recommended to question patients on adherence as deliberate non-adherence could be an indicator of low PIF, this being especially useful when there is a lack of resources to measure PIF.

#### The agreement between ‘critical’ inhalation technique errors from TAI-12 and from the video recordings using checklists

The agreement between in-person inhalation technique assessment by the HCP (TAI-12) and the standardised assessment of video recordings (focussing on errors ‘Teeth and lips sealed around mouthpiece incorrect’; ‘Breathe in incorrect’; ‘Breathing out calmly after inhalation incorrect’)^2^ by two trained researchers was low: 54% agreement (Table 3).

**Table 3:** Agreement between clinician reported inhalation errors (TAI-12) and from video recording assessment.

*n* (%)	From video		
From TAI-12	No error	With error(s)	Total
No error	407 (52.1)	374 (47.9)	781 (54.5)
With error(s)	286 (43.8)	367 (56.2)	653 (45.5)
Total	693 (48.3)	741 (51.7)	1434 (100)

In order to detect ‘critical’ inhalation technique errors, in-person observation was found to be inferior to video rating based on standardised device-specific checklists. Where video assessments are not possible, it is recommended that clinicians should be trained to improve their ability to identify ‘critical’ errors namely ‘teeth and lips sealed around mouthpiece’, ‘breathe in’ and ‘breathing out calmly after inhalation’.

## Discussion

This study provided insight into the substantial proportion of patients with COPD with insufficient inspiratory flow for their DPI, 29% in a stable condition potentially rising to a possible 53% in case of exacerbations. The first clinical recommendation regarding device selection to improve health status of patients with COPD on DPI maintenance therapy in primary care, would be to measure PIF, in addition to observing patient inhaler technique. As sPIF is associated with poorer health status, patients with insufficient inspiratory ability should be switched to an alternative inhaler better suited to the patient’s inspiratory ability, especially if the patient had a higher exacerbation risk. The alternative device, if a DPI, should be checked to see if the patient can generate the optimal flow required. Questioning regarding adherence is informative since deliberate non-adherence is associated with a PIF below minimum for device operation. In case of deliberate non-adherence, we recommend observing the inhalation manoeuvre with the view to correcting critical errors and switching the device. Finally, clinicians should be trained to accurately detect the specific ‘critical’ inhalation technique errors in patients with COPD using a DPI. If possible, recording the inhalation technique and scoring afterwards is optimal to improve the accuracy of the assessment.

We observed that especially sPIF, and to a lesser extent the inhalation technique error ‘Breathe in’, were associated with worse health status. These results are in agreement with a previous observational study that found sPIF to be predictive of all-cause and COPD-related hospital readmission in COPD patients^6^. sPIF could be partially accurately observed, but our findings indicate that objective PIF measurements can provide a better evaluation of a patient’s ability to use and benefit from a DPI. In the PIFotal study, insufficient inspiratory effort has been marked as a critical error for patients with COPD using a DPI. This finding is consistent with evidence from the CRITIKAL study, where this error was found to be associated with uncontrolled symptom control and increased exacerbation rate in patients with asthma^14^. The PIFotal study underlines the need for objective PIF measurements in clinical settings, especially in primary care where these measurements are currently scarce due to equipment and time constraints. However, the In-Check DIAL G16 could be considered a simple method of inhalation technique training, with relatively low one-off costs of around €50, excluding disposable mouthpieces. When objective measurements are not feasible, HCPs could consider patients factors and disease characteristics as determinants of PIF (such as older age, female gender, frailty)^15^ and their adherence to identify the most suitable device for their patients^15^.

Deliberately non-adherent patients were almost twice as likely to have a PIF below the minimum level required for their DPI compared to those who were adherent (OR 1.94, 95% CI [1.26, 3.00] *p* = <0.01, Table 2). This finding is in line with a previous study^8^ and might be explained by limited treatment efficacy in the case of low PIF. We hypothesize a potential vicious circle; where low PIF might lead to insufficient treatment dose into the lungs and thus limited treatment efficacy, which could result in deliberate non-adherence. Subsequently, the non-adherence might hamper a clinical response to the prescribed treatment regimen, worsen the health status, and even lead to a further reduction of PIF. However, the direction of this association could not be established with our cross-sectional study design. Although we need to be cautious when interpreting these findings, the association between deliberate non-adherence and low PIF further emphasizes the importance of PIF measurements in clinical practice.

Finally, we found that the interpretation of ‘critical’ inhalation technique errors differed widely between the in-person assessment by the HCP during the study visit, and the standardised assessment of the video recordings. The inhalation technique in the PIFotal study was recorded and subsequently assessed offline, evaluated by two independent researchers, and a consensus meeting (with a third expert) was held if needed. The approach of recording the inhalation manoeuvre allowed to pause and replay the video, which increased the focus on the inhalation technique details which are usually hard to assess during a consultation. In this way, any potential nuance in the inhalation technique can be found and be scored in more detail, especially considering that a typical inhalation manoeuvre takes place in less than five seconds. Aside from that, the video recordings could be used as a tool to improve the accuracy of error detection. The device-specific checklists used in this study would be feasible for clinical practice, with no costs and with minimal time required to ensure that all inhaler technique steps – required for delivery of the medication – are evaluated and corrected if needed. The feasibility and effectiveness of in-person observation of (dry powder) inhaler technique has been confirmed in patients with asthma. An intervention conducted in community pharmacies, targeting inhaler technique errors with a brief training based on standardized inhaler technique checklists, significantly improved inhaler technique and asthma outcomes^16^.

The strengths of PIFotal include the real-world design, the multinational character of the study, and our large sample of participants with COPD. This allowed us to study a wide range of DPI devices that contributes to the external validity of our findings. The robustness of the PIFotal analysis, and practical recommendations, will help HCPs improve the management of COPD.

Since this was a cross-sectional study, the direction of associations cannot be established. Furthermore, caution is needed when interpreting associations between sPIF and outcomes as PIF is a marker of muscle strength and disease severity. Although we adjusted for a comprehensive set of potential confounders, including disease severity, residual confounding might be present.

Although the international approach is considered a strength of PIFotal, and study procedures were carried out following standardized protocols, the multi-country setting may have introduced heterogeneity in our data. Given our decision tree (Fig. 2), it should be acknowledged that matching the DPI to the patients’ needs should not be solely based on PIF, but that (among others) shared decision-making regarding inhalers^17^, quality of disease control and inhalation technique are factors to consider^5^.

We provide clinical recommendations for primary care clinicians to improve their care of COPD patients by limiting the potential negative consequences of mismatching patients with inhalers. A substantial proportion of COPD patients with insufficient inspiratory flow could benefit from training targeting their peak inspiratory flow, or switching to an alternative DPI better suited to the patient’s inspiratory ability, or active devices such as pMDIs or an SMI. Objective PIF measurements (against the resistance of the patient’s DPI), rather than inspiratory manoeuvre observation ideally should guide the DPI selection process in primary care. HCPs should regularly evaluate the patient’s adherence, as deliberate non-adherence was associated with low PIF. Lastly, we concluded that HCPs should be trained to improve the ability to identify ‘critical’ inhalation technique errors in patients with COPD using a DPI.

## Supplementary information

Supplementary Information

REPORTING SUMMARY

## Data Availability

The datasets generated during and/or analysed during the current study are available from the corresponding author (JK) on request.
