# Supplementary material for: Clinical recommendations for dry powder inhaler use in the management of COPD in primary care
Source: NPJ Prim Care Respir Med. 2022 Dec 27;32:59. doi: 10.1038/s41533-022-00318-3 (PMC9794707; doi:10.1038/s41533-022-00318-3)
Supplement: Supplementary file 1 — Supplementary Information [file 41533_2022_318_MOESM1_ESM.pdf]

## Supplementary Information

**Supplementary Figure 1**: Description of the study steps of the PIFotal study[1]

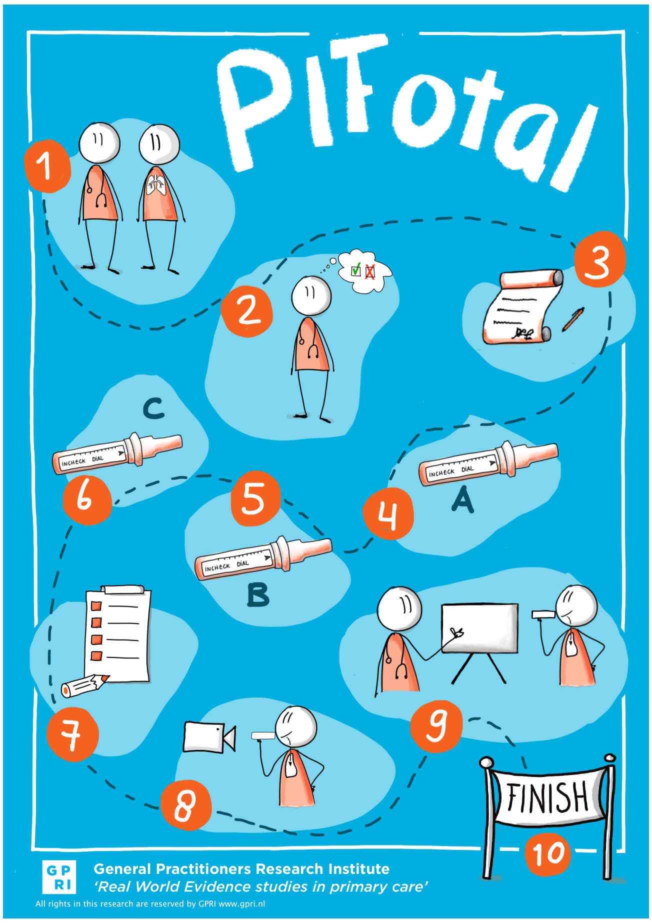


*(1) Participants were invited for clinical examination. (2) Eligiblity was verified. (3) Participants provided written informed consent. (4-6) typical PIF – maximal PIF – maximal PIF at low resistance was assessed. (7) Participants filled out questionnaires to assess health status, number of exacerbations, self-reported medication adherence, medication use, and demographic and clinical covariates. (8) Participants inhaled their usual medication, which was video recorded for offline assessment by two trained researchers based on device-specific checklists. (9) Participants received tailored inhalation instructions based on the inhalation errors they made. (10) The clinical assessment was finished.*

**Supplementary Figure 2.** Flowchart of study population selection.

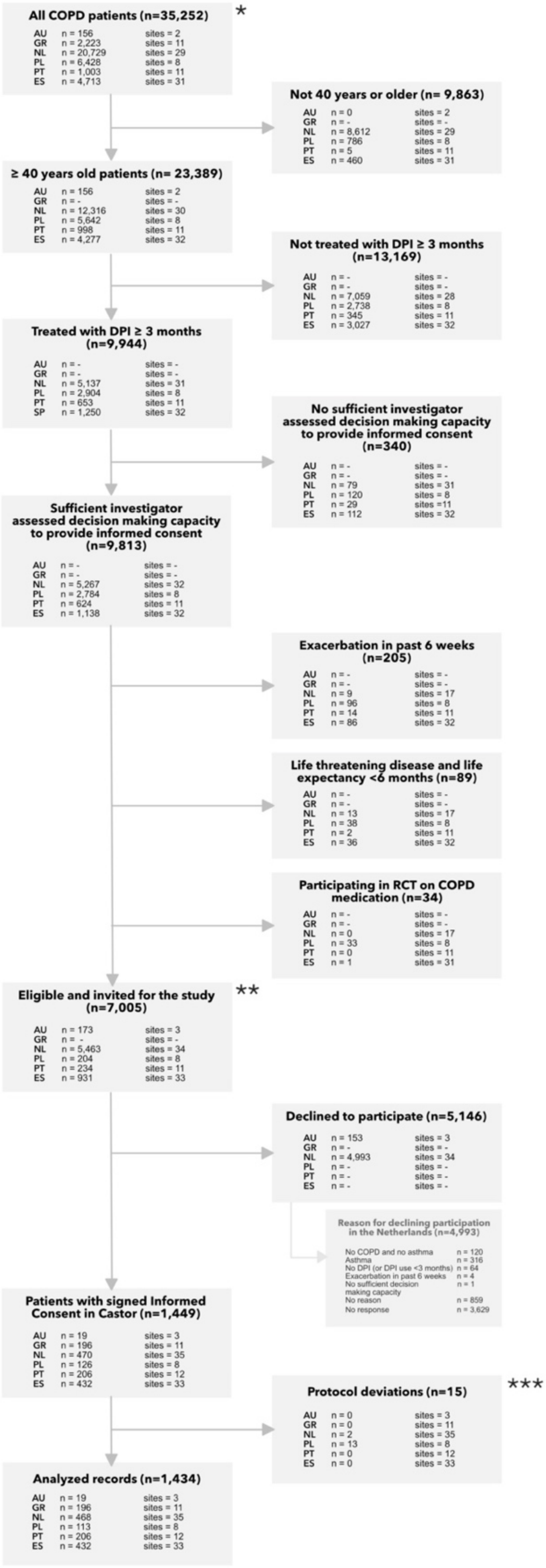


** The data provided by the community pharmacies from Spain are estimations. The data from Greece are also estimations and were calculated before the COVID-19 pandemic. Three sites in Portugal reported relatively low numbers of COPD patients as the sites are specialized care units.*
*** Not all eligible patients in Spain, Portugal and Poland were invited to participate due to time constraints.*
**** These records were archived because the measurements deviated from the study protocol.*

**Supplementary Table 1.** Overview of Dry Powder Inhalers included in the study, the assessment priority for this study and an overview of Peak Inspiratory Flow minimal required / optimal for effective inhalation.

| Inhaler type | Assessment priority | Internal device resistance | Minimal PIF required (L/min) | Optimal PIF (L/min) |
|---|---|---|---|---|
| Ellipta© | 1 | Medium Low | 30 | 60[19,20] |
| Turbuhaler© | 2 | Medium | 30 | 60[19–21] |
| Breezhaler© | 3 | Low | 50 | 50[19,20] |
| Zonda© | 4 | High | 20 | 39* |
| Genuair© | 5 | Medium | 40 | 45[19,20] |
| Novolizer© | 6 | Medium | 35 | 50[19,20] |
| Spiromax© | 7 | Medium | 40 | 40[19,20] |
| Diskus© | 8 | Medium Low | 30 | 60[19–22] |
| Handihaler© | 9 | High | 20 | 30[19,20] |
| Nexthaler© | 10 | Medium High | 35 | 35[19,20] |
| Cyclohaler© | 11 | Low | 40 | 65[19] |
| Easyhaler© | 12 | Medium High | 30 | 30[19,20] |
| Forspiro© | 13 | Medium | 30 | 60[19,20] |
| Elpenhaler© | 14 | Medium Low | 30 | 60** |
| Clickhaler© | 15 | Medium | 15 | 15[23] |

[19]Ghosh, S., Pleasants, R. A., Ohar, J. A., Donohue, J. F. & Drummond, M. B. Prevalence and factors associated with suboptimal peak inspiratory flow rates in COPD. Int. J. COPD 14, 585–595 (2019).

[20]van der Palen, J. Peak inspiratory flow through Diskus and Turbuhaler, measured by means of a peak inspiratory flow meter (In-Check DIAL ®). Respir. Med. (2003) doi:10.1053/rmed.2003.1289.

[21]Clark, A. R., Weers, J. G. & Dhand, R. The Confusing World of Dry Powder Inhalers: It Is All About Inspiratory Pressures, Not Inspiratory Flow Rates. J. Aerosol Med. Pulm. Drug Deliv. 33, 1–11 (2020).

[22]Virchow, J. C., Weuthen, T., Harmer, Q. J. & Jones, S. Identifying the features of an easy-to-use and intuitive dry powder inhaler for asthma and chronic obstructive pulmonary disease therapy: Results from a 28-day device handling study, and an airflow resistance study. Expert Opin. Drug Deliv. (2014) doi:10.1517/17425247.2014.949236.

[23]Newhouse MT, Nantel NP, Chambers CB, Pratt B, Parry-Billings M. Clickhaler (a novel dry powder inhaler) provides similar bronchodilation to pressurized metered-dose inhaler, even at low flow rates. Chest. 115(4), 952–6 (1999).

*Correspondence with manufacturer TEVA, Nov 18 2020.

**Correspondence with Paul Hagedoorn, Head of Inhalation Research Laboratory, RUG.

**Supplementary Table 2.** Inhalation technique errors

| Inhalation step category* | Error designation |
|---|---|
| Preparation of the device incorrect | **Preparation** |
| No removal of the protective cap | **Remove protective cap** |
| Patient did not sit up/stand straight, or head was not tilted such that chin was slightly upwards | **Sit up/stand straight & tilt head** |
| Patient did not hold the inhaler in the correct position during preparation | **Hold inhaler in correct position during preparation** |
| Patient did not hold inhaler in the correct position during the inhalation | **Hold inhaler in correct position during inhalation** |
| Patient did not breathe out to empty lungs before inhalation | **Breathe out completely before inhalation** |
| Patient did not seal teeth and lips around the mouthpiece | **Teeth and lips sealed around mouthpiece** |
| Patient's mouth is not empty. | **Empty mouth before inhalation** |
| Patient did not inhale either strong and deep, or calm and deep (device-specific) | **Breathe in** |
| No breath-hold following the inhalation manoeuvre (or holds breath <6 seconds) | **Hold breath** |
| Patient did not breathe out calmly after inhalation | **Breathe out calmly after inhalation** |
| Patient did not rinse mouth briefly with water. | **Rinse mouth** |

*Adjusted from LAN. Netherlands Lung Alliance (www.inhalatorgebruik.nl)

**Supplementary Table 3.** Overview of confounder candidates for the different associations

**All models:**

| Variable | | Total (N=1434) |
|---|---|---|
| **Country of residence** | Australia, n (%) | 19 (1.3) |
| | Spain, n (%) | 432 (30.1) |
| | Greece, n (%) | 196 (13.7) |
| | The Netherlands, n (%) | 468 (32.6) |
| | Poland, n (%) | 113 (7.9) |
| | Portugal, n (%) | 206 (14.4) |
| **Age** | Mean (SD) | 69.2 (9.3) |
| **Sex** | Female, n (%) | 718 (50.1) |
| | Male, n (%) | 716 (49.9) |
| **Body Mass Index** | Mean (SD) | 27.8 (5.3) |
| **Smoking status** | Current, n (%) | 436 (30.4) |
| | Former, n (%) | 824 (57.5) |
| | Never, n (%) | 174 (12.1) |
| **Educational level** | Primary, n (%) | 436 (30.4) |
| | Secondary, n (%) | 301 (21.0) |
| | Post-secondary vocational, n (%) | 368 (25.7) |
| | University, n (%) | 327 (22.8) |
| **Medication class in device** | LABA, n (%) | 112 (7.8) |
| | LAMA, n (%) | 385 (26.8) |
| | LABA/LAMA, n (%) | 357 (24.9) |
| | LABA/LAMA/ICS, n (%) | 63 (4.4) |
| | ICS, n (%) | 9 (0.6) |
| | ICS/LABA, n (%) | 506 (35.3) |

| | | |
|---|---|---|
| | Short-acting, n (%) | 2 (0.1) |
| **Overall COPD regimen** | Triple Therapy, n (%)<br><br>ICS + (LAMA or LABA), n (%)<br><br>LAMA+LABA, n (%)<br><br>LAMA or LABA or ICS mono, n (%) | 331 (23.1)<br><br>419 (29.2)<br><br>359 (25.0)<br><br>325 (22.7) |
| **SARS-COV-2 history** | Negative, n (%)<br><br>Positive - Managed at home, n (%)<br><br>Positive - Managed at hospital, n (%) | 1353 (95.1)<br><br>56 (3.9)<br><br>13 (0.9) |
| **Lung comorbidity** | n (%) | 122 (8.5) |
| **Cardiovascular comorbidity** | n (%) | 642 (45.0) |
| **Depression** | n (%) | 288 (20.2) |
| **Anxiety** | n (%) | 344 (24.1) |
| **Diabetes mellitus** | n (%) | 295 (20.7) |

**Specific for predictor *Inhalation Quality (combination of PIF and error 'Breathe in'* (RQ2):**

- Non-adherence
- All inhalation errors (except error 'Breathe in')

**Specific for predictor *Deliberate non-adherence* (RQ 3):**

- All inhalation errors (except error related to PIF: 'Breathe in' incorrect)

**Supplementary Table 4.** Inhalation effort with and without exacerbation correction on Peak Inspiratory Flow

| | After exacerbation reduction of 20% of PIFs | | | |
|---|---|---|---|---|
| **Normal classification** | **Can and will do** | **Can, but will not do** | **Cannot do** | **Total** |
| Can and will do | 651 (66.0) | 184 (18.6) | 152 (15.4) | 987 (71.1) |
| Can, but will not do | | 84 (38.5) | 134 (61.5) | 218 (15.7) |
| Cannot do | | | 184 (100.0) | 184 (13.2) |
| Total | 651 (46.9) | 268 (19.3) | 470 (33.8) | 1,389 |
